# Evaluating Opportunities for Achieving Cost Efficiencies Through the Introduction of PrePex Device Male Circumcision in Adult VMMC Programs in Zambia and Zimbabwe

**DOI:** 10.1097/QAI.0000000000000722

**Published:** 2016-05-24

**Authors:** Lyndsey Vandament, Naminga Chintu, Nanako Yano, Owen Mugurungi, Bushimbwa Tambatamba, Gertrude Ncube, Sinokuthemba Xaba, Felton Mpasela, Edward Muguza, Tichakunda Mangono, Ngonidzashe Madidi, Alick Samona, Elva Tagar, Karin Hatzold

**Affiliations:** * Clinton Health Access Initiative, Pretoria, South Africa;; † Society for Family Health (Population Services International), Zambia;; ‡ Population Services International;; § Clinton Health Access Initiative, Lilongwe, Malawi;; ‖ Ministry of Health and Child Care, Harare, Lusaka, Zimbabwe;; ¶ Ministry of Community Development Mother and Child Health, Lusaka, Zambia;; # Clinton Health Access Initiative, Lusaka, Zambia;; ** Clinton Health Access Initiative, Harare, Zimbabwe; and; †† Clinton Health Access Initiative, New York, NY.

**Keywords:** cost, VMMC devices, Zambia, Zimbabwe, integrated service delivery, service delivery model

## Abstract

**Background:**

Results from recent costing studies have put into question potential Voluntary Medical Male Circumcision (VMMC) cost savings with the introduction of the PrePex device.

**Methods:**

We evaluated the cost drivers and the overall unit cost of VMMC for a variety of service delivery models providing either surgical VMMC or both PrePex and surgery using current program data in Zimbabwe and Zambia. In Zimbabwe, 3 hypothetical PrePex only models were also included. For all models, clients aged 18 years and older were assumed to be medically eligible for PrePex and uptake was based on current program data from sites providing both methods. Direct costs included costs for consumables, including surgical VMMC kits for the forceps-guided method, device (US $12), human resources, demand creation, supply chain, waste management, training, and transport.

**Results:**

Results for both countries suggest limited potential for PrePex to generate cost savings when adding the device to current surgical service delivery models. However, results for the hypothetical rural Integrated PrePex model in Zimbabwe suggest the potential for material unit cost savings (US $35 per VMMC vs. US $65–69 for existing surgical models).

**Conclusions:**

This analysis illustrates that models designed to leverage PrePex's advantages, namely the potential for integrating services in rural clinics and less stringent infrastructure requirements, may present opportunities for improved cost efficiency and service integration. Countries seeking to scale up VMMC in rural settings might consider integrating PrePex only MC services at the primary health care level to reduce costs while also increasing VMMC access and coverage.

## Abstract 2

Supplemental Digital Content is Available in the Text.

## BACKGROUND

Achieving the ambitious Voluntary Medical Male Circumcision (VMMC) scale up targets set by Zambia and Zimbabwe in the face of human resource shortages and expected funding declines will require increased efficiency. The PrePex device has the potential to reduce procedure time and increase VMMC output for a given level of staffing. The PrePex may also reduce staff costs by expanding the pool of VMMC providers to more junior nurse cadres with lower salaries. Evidence from PrePex costing and cost comparison studies suggest that reduced procedure times do not always translate into reduced procedure costs because of limited clinical eligibility, low capacity utilization,^1,2^ and higher consumable costs as compared with surgical VMMC.^3^ Existing evidence derives from PrePex clinical trials and from field and pilot implementation studies at research sites; results generated in these settings may not be representative because of higher staffing levels, higher salary costs, and lower capacity utilization in research settings. This study seeks to model the cost efficiency of the PrePex device for current service delivery models used in Zambia and Zimbabwe (Fixed Site, Short Distance Outreach, Long Distance Outreach) and by assessing 3 *hypothetical* “PrePex only” models being developed in Zimbabwe.

## METHODS

### Costing Model and Methodology

The unit cost of VMMC for all service delivery models was calculated in USD using Microsoft Excel 2010 (Microsoft Corporation, Redmond, WA). Unit costs comprise human resources, commodities, training, travel, supply chain, waste management (waste management included for Zimbabwe only as Zambia uses reusable surgical supplies), and demand creation. Program monitoring and management costs were not considered because they are unlikely to vary by circumcision method or model.

For human resources (HR) costs, monthly salaries of public sector and NGO staff were used to estimate annual salaries and calculate daily salary costs (assuming 220 days per annum). Travel allowances applicable for outreach models were also included.

Commodity costs in Zambia were based on consumption data collected for both surgical and PrePex-based circumcisions during the PrePex safety and acceptability study in Q4 2013. In Zimbabwe, commodity costs were based on an adapted version of estimates from the Accelerated Strategic and Operational Plan 2014–2018. The PrePex device cost in both countries was assumed to be US $12 (current procurement price in Zimbabwe).

Training costs per trainee were US $1555 for surgical and US $972 for PrePex in Zambia, and US $795 for a combined training covering both methods in Zimbabwe. Higher costs in Zambia were driven by a longer training schedule for surgical VMMC (8 vs. 5 days) and higher attendee allowances. Training costs were amortized over the number of MCs expected to be performed by each trainee for the service delivery model being costed.

Travel costs for each model were calculated based on average distance traveled and assumed fuel costs in each country (US $0.40/km in Zambia and US $0.19/km in Zimbabwe).

Demand creation costs include personnel costs for interpersonal communication (IPC). Mass media costs were not included. Costs in Zambia were based on monthly Health Promoter salaries (KR 400, ∼US $80) divided by average monthly VMMCs generated. In Zimbabwe, local demand creation agents are paid US $6 per VMMC; thus, the flat payment of US $6 was used.

Unit costs per VMMC for all existing models were calculated based on historical daily output volumes. Data are from March to October 2012 in Zambia and October 2013 to September 2014 in Zimbabwe. For the 3 hypothetical models in Zimbabwe, output scenarios were developed by program staff based on historical experience and sensitivity analysis was used to assess the impact of these assumptions on results. The minimum age for PrePex was assumed to be 18 years, representing 45% and 35% of VMMC volumes' clients in Zambia and Zimbabwe, respectively. An assumed 93% of adults were medically eligible.^4^

### Service Delivery Models

*Current* models evaluated in Zambia and Zimbabwe fall into 3 categories. The *Fixed Site* model is used in government or dedicated NGO sites providing regular (daily/weekly) services; staff receive no travel allowances, and transport costs are minimal. *Short Distance Outreach* (*urban or rural*) is used in settings whereby a full or partial team from a fixed site travels on a weekly/monthly basis, requiring allowances for staff and transport fuel costs. *Long Distance Outreach* is used at sites remote enough to require overnight stays (generally 3–5 days); higher allowances for overnight travel and long distance travel costs are incurred.

**FIGURE 1.**
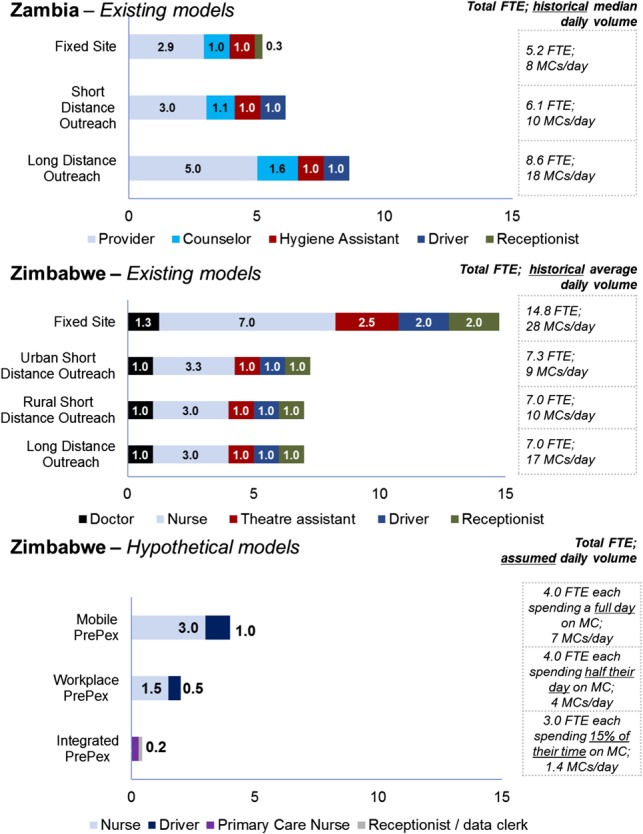
Staffing by service delivery model: number of full time equivalent (FTE) by cadre and service delivery model at a given site on a given day.

Cost assumptions for HR varied for each model according to staffing as illustrated in Figure 1. In Zambia, expected staffing levels were estimated based on the time required for the relevant cadre of staff to complete each procedure. Results were validated as being comparable with actual staffing levels by the Society for Family Health management. In Zimbabwe, data were collected on historical staffing patterns from October 2013 to September 2014 and were also validated by the Population Services International (PSI) management. Transport costs varied by model based on the average distance traveled. Commodity and training costs were not varied by model. However, training cost per VMMC varied based on expected output for each model.

In addition to current service delivery models, 3 hypothetical PrePex only models were evaluated in Zimbabwe. Each was designed to leverage one or more of the benefits of the PrePex procedure: reduced procedure time, client convenience (ability to resume normal activities the same day), removal of sterility requirement, and the use of junior cadre nurses stationed in areas where cadres able to provide surgical VMMC are unavailable. The *Mobile PrePex* model was designed to use a caravan trailer to perform PrePex procedures in high foot traffic areas such as market places and urban residential areas. The *Workplace PrePex* model was designed to reach employees through on-site sensitization sessions and VMMC services during the work day, eg, at lunch hour to minimize disruption to employer and employee. The rural *Integrated PrePex* model was developed for rural sites where primary care nurses (PCNs) provide all services. PrePex VMMC services would be provided by PCNs alongside other health services.

Key assumptions are given as follows:

- A conversion rate of 14% (attendance, and conversion rates are based on the number of men attending workplace sensitizations and the percentage of those attending who subsequently undergo circumcision, as reported through PSI VMMC Workplace Reports from October 2013 to August 2014) was used to estimate the percentage of men reached through IPC who choose to undergo VMMC.
- Of men mobilized for VMMC through Mobile and Workplace models, only 40% were assumed to opt for PrePex as opposed to being referred for surgical VMMC. This is conservative because it is consistent with the uptake rates currently observed at VMMC sites offering both methods, and uptake is likely to increase if PrePex is offered without a surgical option.
- Providers in the Workplace model provide services in a half day, consistent with current workplace outreach conducted by PSI.
- In the rural Integrated PrePex model, providers spend a maximum of 15% of their time on PrePex VMMC.

**FIGURE 2.**
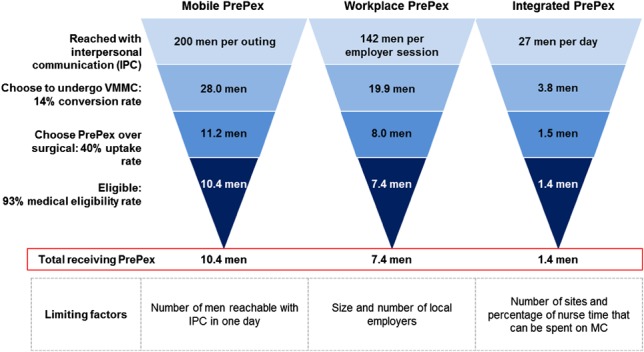
Scenario assumptions for PrePex only models.

Using the above assumptions, the scenarios illustrated in Figure 2 were constructed.

## RESULTS

### Zambia

**FIGURE 3.**
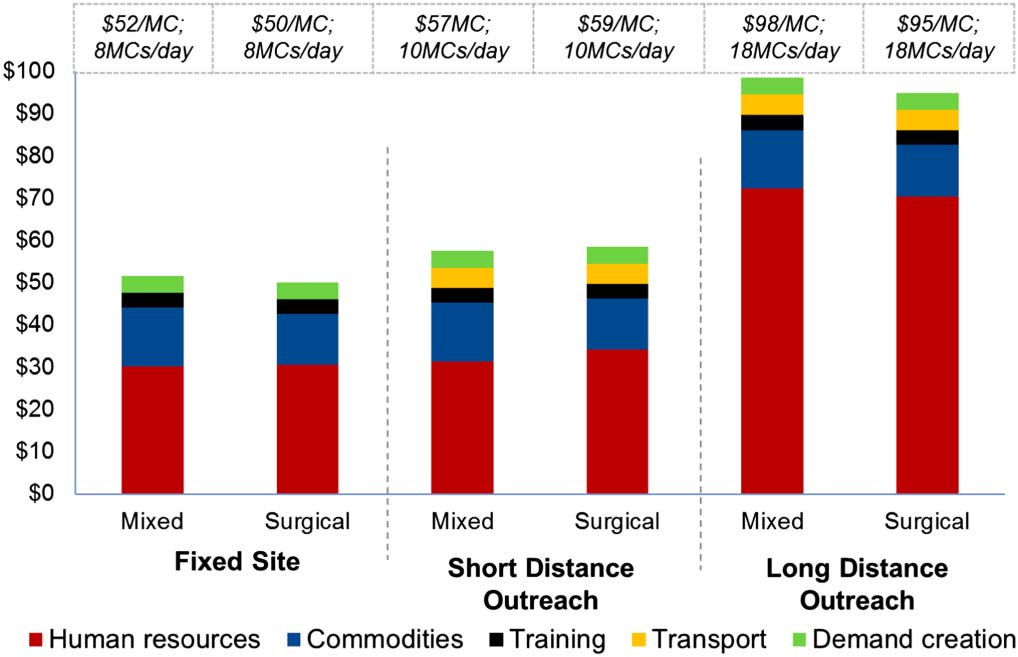
Zambia—average unit costs (US $/VMMC) and median historical daily volumes by service delivery model.

As depicted in Figure 3 and Table S8 (see Supplemental Digital Content, http://links.lww.com/QAI/A704) the unit costs in Zambia for sites providing both dorsal slit surgery and PrePex VMMC (17% PrePex) were US $52 for Fixed Site, US $57 for Short Distance Outreach, and US $98 for Long Distance Outreach. For surgical sites, unit costs were US $50, $59 and $95, respectively. As shown in Table S8 (see Supplemental Digital Content, http://links.lww.com/QAI/A704) the largest cost driver was HR, comprising 54%–74% of costs, respectively. Commodities, including supply chain, comprised 14%–27% of costs. Training, transport, and demand creation costs contributed negligibly to unit cost for all models.

### Zimbabwe

**FIGURE 4.**
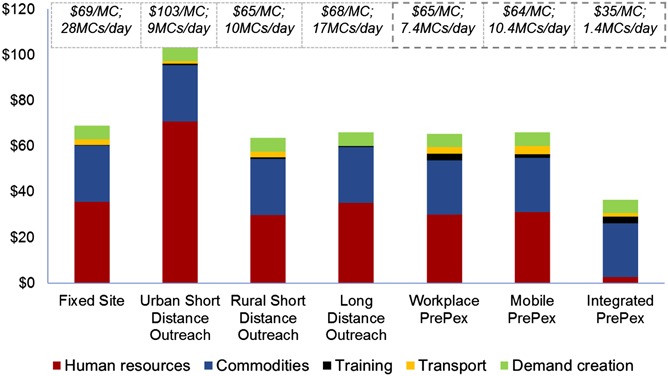
Zimbabwe—average unit costs (US $/VMMC) and historical average daily volumes by service delivery model.

In Zimbabwe, the costs of *current* forceps-guided surgical service delivery models, as defined in Methods section, were compared with those of new hypothetical PrePex only models (Fig. 4; see Table S9, Supplemental Digital Content, http://links.lww.com/QAI/A704). Unit costs per VMMC for current models were US $69 for Fixed Site, US $103 for Urban Short Distance Outreach, US $65 for Rural Short Distance Outreach, and US $68 for Long Distance Outreach. The largest cost driver was HR, comprising 51%, 68%, 46%, and 51%, respectively, of costs due to varying levels of staff utilization. Commodities including supply chain, comprised 35%, 24%, 38%, and 36% of costs, respectively. Training, transport, and demand creation costs contributed negligibly to unit costs for current models.

In comparison, unit costs for the hypothetical PrePex only models were US $65 for Workplace PrePex, US $64 for Mobile PrePex, and US $35 for Integrated PrePex. For Workplace and Mobile models, the largest cost driver was HR constituting 46% and 49% of costs, respectively. Commodities constituted 36% and 37% of costs, respectively. For the Integrated model, commodities comprised 67%, demand creation costs 17%, HR costs 7%, and training costs 8% of total costs. Lower HR costs for integrated PrePex were the result of including only time dedicated to VMMC in HR costs, with unused nurse time assumed to be taken up by other services because of the model's integrated nature.

**FIGURE 5.**
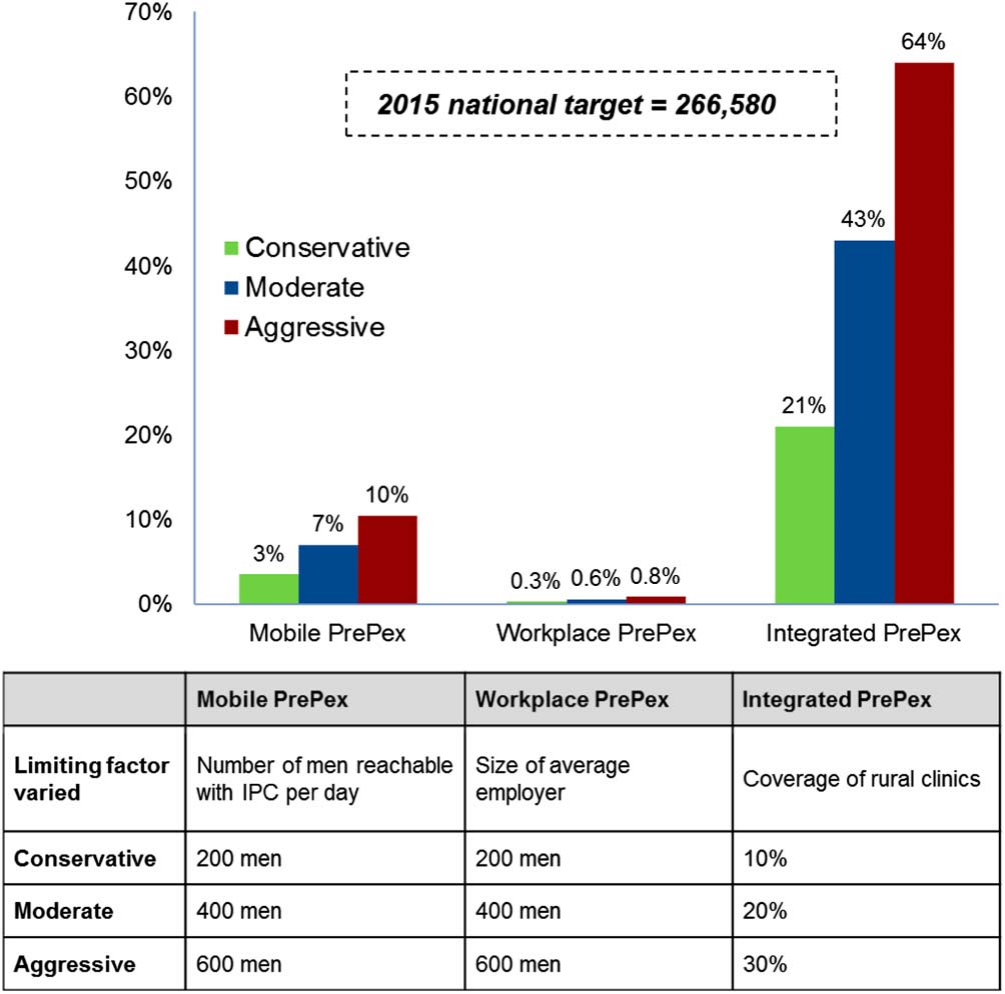
Sensitivity analysis assumptions and potential coverage of 2015 national target for Zimbabwe using PrePex-only models.

The percentage of Zimbabwe's national target achievable through each hypothetical model was estimated using historical program data and assumptions derived from the relevant literature as follows: for Workplace PrePex IPC, reach was based on the number of males formally employed at businesses with more than 200 male employees^5^ and a 71% attendance rate for VMMC sensitization sessions (VMMC volumes for existing models were predominantly surgical, although a limited number of teams began offering PrePex in the second half of the costing period) was assumed. Sensitivity analysis was conducted based on the limiting factors described in Figure 2. Results suggest that an “aggressive” scale-up of Mobile, Workplace, and Integrated PrePex models could achieve 10%, 0.8%, and 64%, respectively, of Zimbabwe's 2015 target (Fig. 5).

## CONCLUSIONS

Unit cost estimates in Zambia suggest immaterial changes in cost with PrePex introduction even at high volumes. The use of low-cost reusable surgical commodities results in PrePex commodities costing ∼US $10 more per VMMC (an increase of ∼US $1.80 per VMMC for mixed sites), based on a device price of US $12, which is less than half the price paid by some programs. In this context, the upfront introduction costs of revising guidelines and demand generation materials, and expanding the supply chain, may exceed future cost savings. Our results do not provide financial justification for PrePex introduction in Zambia for current models.

In Zimbabwe, average unit costs for existing models, except Urban Short Distance Outreach, fall within a narrow range of US $65–US $69. Urban Short Distance Outreach services face higher costs because of required staffing levels and thus are rarely used (Urban Short Distance Outreach constituted only 3% of PSI volumes in Zimbabwe during the study period). The wide variation in average team size (Fig. 1) was aligned to average output, allowing all 3 models to achieve similar cost efficiency.

As PrePex is not expected to increase efficiencies for current models in Zimbabwe,^2^ PrePex only models would warrant introduction if they can achieve unit costs below the “efficient range” of US $65–$69 (Fig. 4) in already serviced geographies, or within this range for models with the potential to increase program coverage. Moreover, these adult-focused models could generate incremental demand in this age group; only 35% of current VMMCs are performed on adults 18 years and older, although adults account for approximately 64% of the target cohort (men, 13–29). Operational research would be required to confirm whether or not PrePex models increase demand for older men.

As illustrated in Figure 4, the Mobile and Workplace unit costs are equal to or are slightly lower than current models. At US $35 per VMMC, the Integrated PrePex model suggests potential for material cost savings. Operational research would be needed to determine the feasibility of PCNs, dedicating 15% of time to VMMC given other demands on their time; however, lower output would reduce total volumes but not efficiency as nurse time would be redirected to other services. The assumption that nurse time is always fully used for VMMC or other services drives the material reduction in HR costs for this model. Mobile and Workplace models rely on dedicated staff and could only reduce costs through increased uptake. Uptake of 60%, instead of the assumed 40%, would reduce unit costs for Mobile and Workplace models to US $52 and US $56, respectively. This analysis differs from previous studies,^2^ which compared the cost efficiency of sites providing both methods to the provision of surgical VMMC only.

Given the low estimated savings and potential coverage (Fig. 5), introduction of Workplace and Mobile PrePex models may only be justifiable if evidence of higher uptake rates emerges, upfront investment costs are minimal, or potential exists to reach high-risk populations otherwise underserved. The Integrated model, however, may achieve net savings if upfront investments are required and may be the most sustainable in the long run as it is integrated into government services. However, implementation would require that surgical VMMC teams and capacity at the district level be maintained to respond to adverse events and provide services to men who require or prefer surgical VMMC. Furthermore, the long-term usefulness of this model would rely on the prequalification of PrePex for men younger than 18 years.

These results suggest PrePex introduction could lead to efficiencies in Zimbabwe, which was not the conclusion reached in Zambia. This is driven by differences in commodity costs, site staffing, and remuneration. In Zimbabwe, commodity costs for both methods are very similar as disposable surgical supplies are used because of limited autoclaving capacity. Furthermore, all cadres of nurses in Zambia are able to provide surgical VMMC (which is not the case in Zimbabwe), and the use of models dedicated to 1 method was not being investigated in Zambia because of a desire to provide clients with their choice of method. Lastly, in Zimbabwe, the remuneration scheme allows for staff providing VMMC services during regular work hours to be reimbursed even when travel is not involved. This is not the case in Zambia and is likely to increase support for decentralized integrated service delivery.

This analysis is limited by our reliance on modeled unit costs. Operational research is needed to confirm these results and determine if there are any incremental costs required to implement these models. Specifically, supply chain costs may change as introducing PrePex and decentralizing services will require stronger coordination and forecasting capabilities to avoid stock outs (particularly of device sizes); supervision costs may be higher for a larger network of rural clinics; equipment costs for caravan vehicles and beds may exceed savings for Workplace and Mobile PrePex models; and referral costs may be necessitated if PrePex uptake is low. Lastly, future operational research should also assess whether offering PrePex generates any incremental demand.

Our analysis illustrates that PrePex models leveraging the potential for integrating services in rural clinics and less stringent infrastructure requirements may improve cost efficiency and service integration. However, opportunities need to be evaluated on a country-by-country basis as the relative efficiency of PrePex models is strongly influenced by national guidelines for the cadres who perform surgical VMMC, staff remuneration schemes, and the surgical commodities used. Furthermore, these conclusions may not extend to devices, such as ShangRing, that require sterility and are considered surgical.

## References

[1] Duffy K Galukande M Wooding N Reach and cost-effectiveness of the PrePex device for safe male circumcision in Uganda. PLoS One. 2013;8:e63134. 23717402 10.1371/journal.pone.0063134 PMC3661578

[2] Njeuhmeli E Kripke K Hatzold K Cost analysis of integrating the PrePex medical device into a voluntary medical male circumcision program in Zimbabwe. PLoS One. 2014;9:e82533. 24801515 10.1371/journal.pone.0082533 PMC4011574

[3] Obiero W Young MR Bailey RC The PrePex device is unlikely to achieve cost-savings compared to the forceps-guided method in male circumcision programs in Sub-Saharan Africa. PloS One. 2013;8:e53380. 23349708 10.1371/journal.pone.0053380 PMC3549910

[4] WHO. Guidelines on the Use of Devices for Adult Male Circumcision for HIV Prevention. Geneva, Switzerland, World Health Organization; 2013. 24624479

[5] Luebker M Employment, Unemployment and Informality in Zimbabwe: Concepts and Data for Coherent Policy-Making. SRO-harare Issues Paper No.32/Integration Working Paper, No. 90. International Labour Office, Policy and Integration and Statistical Department. ILO Sub-Region Office for Southern Africa (SRO-Harare). Geneva, Switzerland: ILO; 2008.

